# Malaria-Cutaneous Leishmaniasis Co-infection: Influence on Disease Outcomes and Immune Response

**DOI:** 10.3389/fmicb.2016.00982

**Published:** 2016-06-27

**Authors:** Raquel A. Pinna, Danielle Silva-dos-Santos, Daiana S. Perce-da-Silva, Joseli Oliveira-Ferreira, Dea M. S. Villa-Verde, Paula M. De Luca, Dalma M. Banic

**Affiliations:** ^1^Laboratory of Simulids, Onchocerciasis and Sympatric Diseases: Mansonelliasis and Malaria, Oswaldo Cruz Institute, Oswaldo Cruz FoundationRio de Janeiro, Brazil; ^2^Laboratory of Thymus Research, Oswaldo Cruz Institute, Oswaldo Cruz FoundationRio de Janeiro, Brazil; ^3^Laboratory of Immunoparasitology Research, Oswaldo Cruz Institute, Oswaldo Cruz FoundationRio de Janeiro, Brazil

**Keywords:** co-infection, Malaria, Leishmaniasis, immunology, cytokine, *Plasmodium yoelii*, *Leishmania braziliensis*, *Leishmania amazonensis*

## Abstract

Malaria and Cutaneous Leishmaniasis (CL) are co-endemic throughout large regions in tropical countries and co-infection may impact the evolution of host-parasite interactions. In the present study, we evaluate Malaria/Leishmaniasis disease outcome, Th1/Th2 cytokine levels and the CD4 and CD8 T-cell profiles in a co-infection murine model (BALB/c) of *Plasmodium yoelii* 17XNL (Py) and *Leishmania amazonensis* (La) or *L. braziliensis* (Lb). Malaria parasitaemia was assessed through blood strains stained with Giemsa. *Leishmania* lesions were monitored with a digital caliper and parasite loads determined by limiting-dilution assay. Serum levels of IFN-γ, TNF, IL-2, IL-4, IL-6, IL-10, and IL-17 were determined using multiplexed bead assay and expression of CD3, CD4, and CD8 T-cells markers were determined by Flow Cytometry in the thymus, spleens and lymph nodes. Parasitaemia in Lb+Py co-infected group was lower than in Py single-infected group, suggesting a protective effect of Lb co-infection in Malaria progression. In contrast, La+Py co-infection increased parasitaemia, patent infection and induced mortality in non-lethal Malaria infection. Regarding Leishmaniasis, Lb+Py co-infected group presented smaller lesions and less ulceration than Lb single-infected animals. In contrast, La+Py co-infected group presented only a transitory delay on the development of lesions when compared to La single-infected mice. Decreased levels of IFN-γ, TNF, IL-6, and IL-10 were observed in the serum of co-infected groups, demonstrating a modulation of Malaria immune response by *Leishmania* co-infections. We observed an intense thymic atrophy in Py single-infected and co-infected groups, which recovered earlier in co-infected animals. The CD4 and CD8 T cell profiles in thymus, spleens and lymph nodes did not differ between Py single and co-infected groups, except for a decrease in CD4^+^CD8^+^ T cells which also increased faster in co-infected mice. Our results suggest that Py and *Leishmania* co-infection may change disease outcome. Interestingly Malaria outcome can be altered according to the *Leishmania* specie involved. Alternatively Malaria infection reduced the severity or delayed the onset of leishmanial lesions. These alterations in Malaria and CL development seem to be closely related with changes in the immune response as demonstrated by alteration in serum cytokine levels and thymus/spleens T cell phenotypes dynamics during infection.

## Introduction

Malaria and Cutaneous Leishmaniasis (CL) are two of the world’s most important vector-borne parasitic diseases (World Health Organization, 2010, 2015; Alvar et al., 2012). Malaria, an infectious disease caused by *Plasmodium* genus parasites, is an important cause of global mortality and morbidity. Half of the world population is at risk of contracting Malaria, with approximately 214 million cases and 438 000 deaths in 2015, amongst the 3.2 billion people living at risk of infection (World Health Organization, 2015). Humans can be infected by five *Plasmodium* species: *P*. *falciparum, P. vivax, P. ovale, P. malariae*, and *P. knowlesi*. Although *P. falciparum* accounts for the great majority of morbidity and mortality, *P. vivax* has a wider geographic distribution and causes considerable symptomatic disease (Battle et al., 2014). Malaria infection has a variable clinical phenotype, ranging from a mild febrile illness to severe disease and death, but infection can also occur in the absence of clinical symptoms. These variations in disease pattern are attributable to many factors, including the genetic background of the host and pathogen, the complex relationship between the parasite and host immune response, the dynamics of parasite transmission and/or the biological interactions of the parasites within the host (Good and Doolan, 2010; van den Bogaart et al., 2012). Leishmaniasis is a complex disease caused by different species of intracellular protozoan parasites from the genus *Leishmania*, which also induces significant morbidity and mortality throughout the world. According to the World Health Organization (2016) 350 million people in 98 countries are at risk of infection. There are an estimated annual 1.3 million new cases worldwide, of which 300,000 cases are of Visceral Leishmaniasis (VL) and another 1 million of CL. Seventy (70) to 75% of the CL cases occur in Afghanistan, Algeria, Brazil, Colombia, Costa Rica, Ethiopia, Iran, Peru, Sudan, and Syria (World Health Organization, 2016). CL is caused by *L. major, L. tropica*, and *L. aethiopica* in the Old World, whereas in the New World it is most frequently caused by *L. mexicana, L. amazonensis, L. braziliensis, L. panamensis*, and *L. guyanensis* (World Health Organization, 2010; Hartley et al., 2014). Symptoms range from the more prevalent single self-healing cutaneous lesions to uncontrolled parasite replication, producing non-healing cutaneous, mucosal or even visceral disease as well as chronic metastatic dissemination throughout the skin. This spectrum of manifestations is multifactorial and depends on complex interactions among parasite, host, and environmental factors, including the *Leishmania* specie, genetic background and immunological status of the host (Hartley et al., 2014).

The overlapping geographic distribution of Malaria and Leishmaniasis, especially in the tropical and subtropical countries demonstrate clearly that the potential for interaction among these parasites may occur and play a role in determining disease outcome (Hotez et al., 2006; van den Bogaart et al., 2012, 2014). Despite this natural coexistence, data from concomitant infections are so far not available in the literature (Ab Rahman and Abdullah, 2011; van den Bogaart et al., 2012, 2014). Therefore, the impact of the dual infections on the human population health remains unassessed particularly in what concerns CL.

In the eighties, two studies in the murine model evaluated the effect of non-lethal *P. yoelii* and *L. amazonensis* concomitant infections in the course of each disease (Coleman et al., 1988a,b). These studies demonstrated that severity and susceptibility to both diseases were enhanced during co-infection. Since these parasites do not compete for the same host cells, neither anatomical sites nor resources, the interaction among them is most likely indirect and related mostly to the host’s immune responses induced by each pathogen. However, in both studies (Coleman et al., 1988a,b) the immunological mechanisms involved were not investigated. Alternatively, several studies have shown that outcome of Malaria and CL is determined, in part, by the balance of pro- and anti-inflammatory immune responses (Louis et al., 1998; Li et al., 2001; Silveira et al., 2009; Taylor-Robinson, 2010; Medina et al., 2011; Freitas do Rosario and Langhorne, 2012; Kedzierski and Evans, 2014).

The immune response against the blood stages of Malaria parasites operate in concert and sequentially to control and clear the parasitaemia by an early and strong pro inflammatory, type 1 response (Th1), limiting parasite growth, followed by a shift to anti-inflammatory, type 2 immune (Th2) response. The balance between cytokines produced by pro inflammatory and anti-inflammatory responses during different phases of the blood stage infection determines the outcome of the disease (Li et al., 2001). In contrast to the strong type 1 immune response at the beginning of the Malaria infection, CL caused by *L. amazonensis* generally results in type 2 cellular immune response polarization (Silveira et al., 2009; Gollob et al., 2014). On the other hand, in CL caused by *L. braziliensis*, there is evidence that increased production of inflammatory cytokines (IFN-γ and TNF-α) and absence of IL-10 is associated with tissue destruction and the development of mucosal lesions (Dutra et al., 2011; Gollob et al., 2014; Oliveira et al., 2014). In this context, the current study was designed to evaluate the outcome of Malaria-CL infections, the CD4 and CD8 T-cell profiles as well as the Th1/Th2 cytokine levels in a co-infection murine model (BALB/c) of *P. yoelii* 17XNL (non-lethal) and *L. amazonensis* or *L. braziliensis*. Furthermore, as far as we know, this is the first report utilizing *L. braziliensis* and *P. yoelii* 17XNL non-lethal strain as a co-infection model.

## Materials and Methods

### Animals, Parasites, and Infections

Female BALB/c mice (5–6 weeks old) were obtained from the Center for Laboratory Animal Breeding of the Oswaldo Cruz Foundation (FIOCRUZ) (Rio de Janeiro, RJ, Brazil). Mice were maintained under specific pathogen-free conditions in Experimental Animal Center of Leônidas Deane building (FIOCRUZ). This research protocol was approved by the Ethical Committee for Animal Use of FIOCRUZ/MS (license LW-17/11).

*P. yoelii* 17XNL (non-lethal strain) was provided by Dr Fábio T.M. Costa at Departamento de Genética, Evolução e Bioagentes, Instituto de Biologia, UNICAMP, Campinas, SP, Brazil. Parasite stabilates were stored at -196°C. To obtain the experimental inoculum of *P. yoelii* 17XNL, parasitized red blood cells (pRBCs) were defrosted and passed through three homologous donor mice.

Promastigotes of *L. braziliensis* (MCAN/BR/98/R69) and *L. amazonensis* (IFLA/BR/67/PH8), provided by Laboratório de Pesquisa em Leishmaniose, Instituto Oswaldo Cruz, FIOCRUZ, RJ, Brazil, were cultured in Schneider’s medium supplemented with antibiotics (200 IU penicillin and 200 μg streptomycin/ml) and 10% inactivated fetal calf serum (all from Sigma–Aldrich, St Louis, MO, USA). For *L braziliensis* 2% of sterile human urine was also added to the cultures (Howard et al., 1991).

**FIGURE 1:**
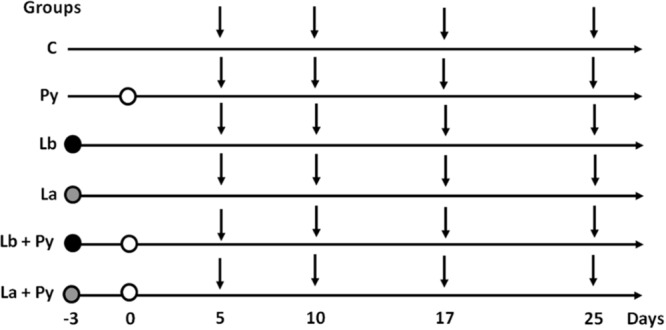
**Experimental design.** Mice were infected with *Leishmania braziliensis*. (black circles) or *L. amazonensis* (gray circles) 3 days before *Plasmodium yoelii* infection (white circles). On days 5, 10, 17, and 25 post *P. yoelii* infection (vertical arrows), six mice from Py, Lb, La, Lb+Py, and La+Py groups and four mice from control (C) group were euthanized to evaluate parasite loads in the ears and draining lymph nodes (Lb/La and Lb+Py/La+Py groups), cytokine serum levels (all groups), thymus relative weight (all groups), absolute numbers of thymocytes (all groups) and phenotyping of T cells in the thymus, spleen and lymph nodes (all groups).

Eight to nine-week-old mice (∼22g each) were divided randomly into six groups, **Figure 1**. Group C: comprised uninfected mice; Group Py: mice were injected intraperitoneally (i.p.) with 10^6^ *P. yoelii* 17XNL pRBCs (0.2 mL); Groups Lb and La: mice were inoculated intradermally in both ears with either 10^5^ or 10^4^ stationary phase promastigotes of *L. braziliensis* or *L. amazonensis*, respectively, as previously described (Belkaid et al., 2000); Groups Lb+Py and La+Py: mice were co-infected with both *P. yoelii* 17XNL and either *L. braziliensis* or *L. amazonensis*. Each group had 25 to 35 mice and experiments were repeated at least three times. First, mice were infected with *Leishmania* sp. and 3 days later with *P. yoelii* 17XNL, as shown in **Figure 1**. At 5, 10, 17, and 25 days post-*P. yoelii* 17XNL infection, six animals from each group were bled, for serum cytokine assay, and euthanized, for thymus, spleen, ears and lymph nodes removal. Mortality of mice was monitored daily, post-*P. yoelii* 17XNL infection.

### Determination of Parasite Load, Leishmania Lesion Size, and Ulceration

Malaria parasitaemia was monitored every 2 days starting at day 3 post-infection. Blood smears were prepared from tail vein, methanol-fixed, stained with Giemsa and microscopically examined to determine parasitaemia in 1,000 erythrocytes. The percentage of infected erythrocytes was calculated as follows: Parasitaemia (%) = (number of infected erythrocytes x 100)/total number of erythrocytes counted (1,000).

The diameter of dermal *Leishmania* sp. lesions were measured weekly with a digital calipter. We also monitored the time elapsed until ulcer formation in nodules. Parasite numbers in the ears and draining lymph nodes were determined as previously described (Belkaid et al., 2000) and scored as the highest dilution containing viable parasites after incubation for 6 days at 26°C.

### Cytokines Quantitation in the Serum

The serum levels of cytokines (interferon – IFN-γ, tumor necrose factor – TNF, interleukin (IL)-2, IL-4, IL-6, IL-10, and IL-17) were determined using the BD Cytometric Bead Array Kit Mouse Th1/Th2/Th17 Cytokines (BD Biosciences, San Jose, CA, USA) according to the manufacturer’s instructions. Briefly, 25 μL of mouse serum was incubated for 2 h at room temperature with 25 μL of cytokine capture bead and 25 μL of phycoerythrin (PE) detection reagent. Then, samples were washed with 1 mL of buffer by centrifugation (200 × *g*, 5 min). The supernatants were carefully aspirated and discarded from each assay tube. Finally, beads were resuspended in 300 μL of buffer for analysis on a FACSCalibur flow cytometer (Becton Dickinson, San Jose, CA, USA). The serum cytokine concentrations (pg/mL) were determined using a standard curve from recombinant cytokines provided by the kit.

### Determination of Thymus Relative Weight

For determination of thymus relative weight, body and thymus of each mouse were weighted. Thymic index (TI) was calculated according to the following formula: TI = thymus weight (mg) / body weight (g).

### T Cell Phenotyping by Flow Cytometry

Thymus, spleens, mesenteric nodes, subcutaneous nodes (cervical, axillary, brachial, and inguinal) and submandibular nodes (draining ears lesions) were dissected and mechanically disaggregated. Single-cell suspensions from control, infected and co-infected mice were obtained in RPMI-1640 supplemented with 10% fetal calf serum (Gibco-Invitrogen, USA). Cell numbers and viability were determined by Trypan blue exclusion using Neubauer chamber.

For T lymphocytes phenotypic characterization, 10^6^ cells were stained with FITC labeled anti-mouse CD4, APC labeled anti- mouse CD8 and PE labeled anti-mouse CD3 monoclonal antibodies. After incubation for 30 min, followed by 2 washes with staining buffer (PBS, 0,1% BSA, 0,01% sodium azide, all from Sigma–Aldrich), cells were fixed with 1% paraformaldehyde solution (Sigma–Aldrich), washed and resuspended in staining buffer until acquisition. A minimum of 30,000 events per sample were acquired inside the lymphocytes gate, based on size and granularity properties using a FACSCantoII^TM^ flow cytometer (BD Bioscience, USA) and analyzed using FlowJo 7.5.5 software (Tree Star Inc. Ashland, OR, USA).

### Statistical Analysis

Sample size was determined *a priori* using the software G*Power version 3.1.9.2, based on the Mann–Whitney test, a significance level of 0.05, and a minimum power of 80%. Results were evaluated by a non-parametric test, the Mann–Whitney test, using GraphPad Prism software version 5.0 (GraphPad Software, La Jolla, CA, USA). Data are expressed as means ± SEM and considered to be statistically significant when *p* < 0.05. Survival curves were analyzed by Mantel Cox and Gehan–Breslow–Wilcoxon tests and *Leishmania* lesions ulcerations were analyzed by Chi-square test using the same software.

## Results

### *L. braziliensis* and *L. amazonensis* co-Infection exerts opposite effects on *P. yoelii* 17XNL Infection

**FIGURE 2:**
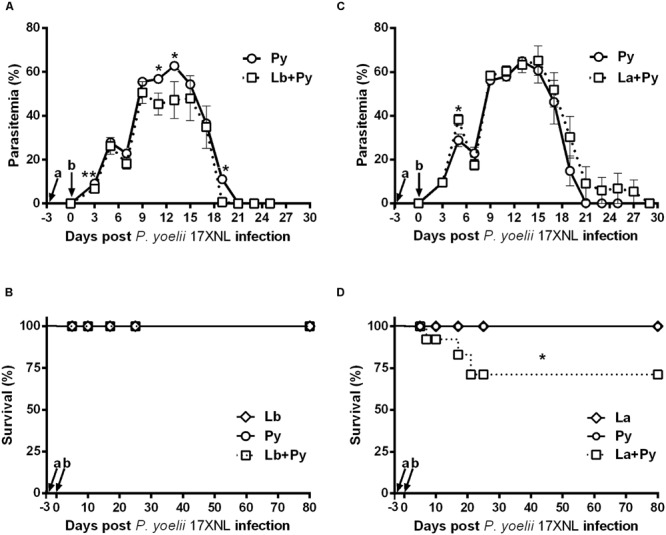
**Parasitaemia and survival rate of BALB/c mice infected with blood-stage *P. yoelli* 17XNL non-lethal strain and co-infected with *Leishmania* sp. and *P. yoelii* 17XNL. (A,C)** Malaria parasitaemia in *P. yoelii* single-infected mice (Py) and in mice co-infected with *L. braziliensis/P. yoelii* (Lb+Py) (A) or with *L. amazonensis/P. yoelii* (La+Py) **(C)**. **(B,D)** Comparison of the survival rate in Py single-infected and Lb+Py co-infected **(B)** or La+Py co-infected mice **(D)**. Parasitaemia results are expressed by the mean ± SEM of at least 10 mice per group. The Mann–Whitney test was used to compare Py single-infected and co-infected groups. Survival rates were compared by Mantel-Cox test. ^∗^*p* < 0.05 and ^∗∗^*p* < 0.01, respectively. Arrows indicate the time of *Leishmania* (a) and *P. yoelii* (b) infection.

Blood stage *P. yoelii* 17XNL parasites induced Malaria infection in all BALB/c mice from single-infected (Py) and *L. braziliensis* or *L. amazonensis* co-infected groups (Lb+Py and La+Py). Parasitaemia peak occurred between 10 and 17 days after infection and no parasite was detected after 23 days post-infection in blood smears of Py single-infected mice. The overall mean parasitaemia observed in Lb+Py co-infected group was lower than that observed in *P. yoelii* single-infected group (Py). Statistically significant differences observed in some days are suggestive for a protective effect of *L. braziliensis* co-infection in Malaria progression (**Figure 2A**). In contrast, co-infection with *L. amazonensis* appears to have a negative influence in acute Malaria (**Figure 2C**), and mortality in La+Py co-infected group enhanced in comparison to Py single infected group (**Figure 2D**). La+Py group exhibited higher parasitaemia on day 5 and a longer course of *P. yoelii* Malaria patency with mice remaining parasitemic 8 days longer than mice in Py single-infected group (**Figure 2C**). The higher rate of parasitemia in La+Py coinfeted mice seems to be associated with decreased survival. Four weeks after *P. yoelii* 17XNL infection, 30% of the co-infected La+Py mice were dead, compared to 100% of survival in the group infected only with *P. yoelii* 17XNL. Py single-infected groups showed no mortality during the whole experiment (**Figures 2B,D**).

### Co-Infection is Able to Reduce Severity of *L. braziliensis* Lesions

**FIGURE 3:**
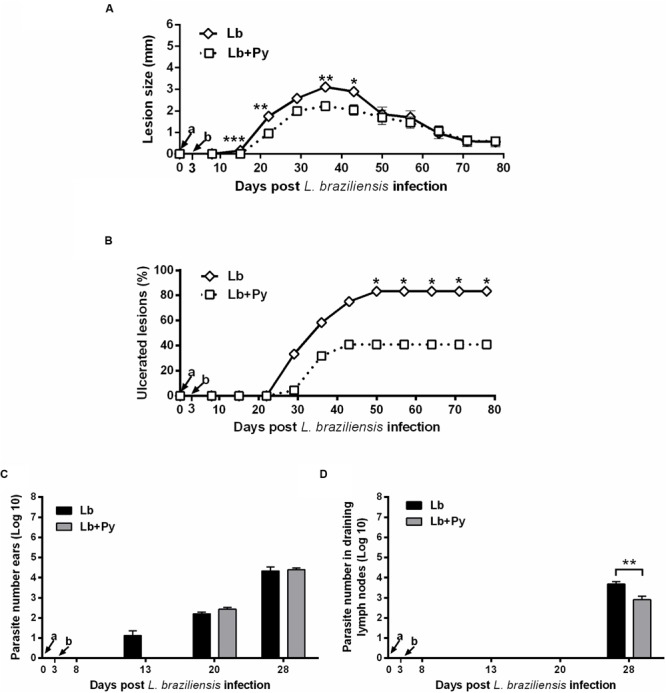
**Evaluation of *L. braziliensis* infection. (A)** Lesion development, **(B)** onset of open ulcers, and **(C)** parasite quantitation in infected ears and **(D)** draining lymph nodes of BALB/c mice after intradermal infection in the ears with *L. braziliensis* promastigotes (Lb) or co-infected with *L. braziliensis* and *P. yoelii* (Lb+Py). Parasite loads were individually determined by a limited dilution assay. Results are expressed by the mean ± SEM of twelve ears and lymph nodes. The Mann–Whitney test was used to compare lesion size **(A)** and parasite loads in ears **(C)** and draining lymph nodes **(D)**. Chi-square test was used to evaluate ulcerations **(B)**. ^∗^*p* < 0.05, ^∗∗^*p* < 0.01, and ^∗∗∗^*p* < 0.001, respectively. Arrows indicate the time of *L. braziliensi*s (a) and *P. yoelii* (b) infection.

BALB/c mice were intradermally infected with 10^5^ stationary phase promastigotes of *L. braziliensis*. Lb+Py co-infected group showed an overall tendency to present smaller lesion sizes than Lb single infected group (**Figure 3A**). In both groups, lesions reached maximum size 5 weeks post-infection and started to heal afterward. To evaluate disease severity we followed the onset of open ulcers in *Leishmania*-infected and co-infected groups (**Figure 3B**). Ulcers started to appear 21 days post-infection in Lb group and after 28 days in Lb+Py. At week 11 after infection (77 days) Lb single-infected group presented a higher number of ulcerated nodules (83.3%) than Lb+Py (40.9%). At week 17 and 18 post infection, there was a complete resolution of lesions in Lb+Py and Lb groups, respectively (data not shown). Limiting-dilution assays performed in the ears and in draining lymph nodes showed an earlier detection of parasites in the ears of Lb single-infected animals than in co-infected group (14 and 21 days post-infection, respectively) with no difference in parasite loads between groups. On the other hand, in the draining lymph nodes parasites were detected 28 days after infection in both groups, and parasite loads were lower in Lb+Py co-infected group (*p* = 0.006, **Figures 3C,D**).

### Co-Infection Reduce Severity of *L. amazonensis* Lesions Only during Acute Phase Malaria

**FIGURE 4:**
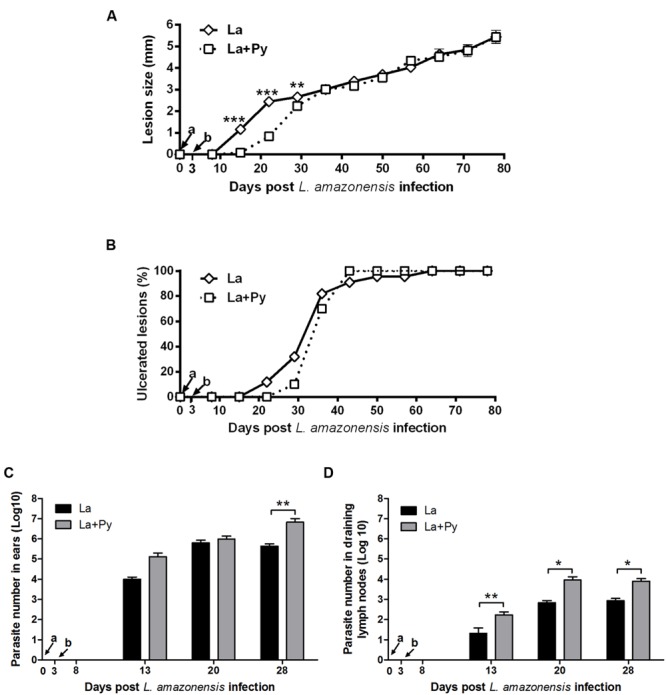
**Evaluation of *L. amazonensis* infection. (A)** Lesion development, **(B)** onset of open ulcers, and **(C)** parasite quantitation in infected ears and **(D)** draining lymph nodes of BALB/c mice after intradermal infection in the ears with *L. amazonensis* promastigotes (La) or co-infected with *L. amazonensis* and *P. yoelii* (La+Py). Parasite loads were individually determined by a limited dilution assay. Results are expressed by the mean ± SEM of twelve ears and lymph nodes. The Mann–Whitney test was used to compare parasite loads in ears and draining lymph nodes. ^∗^*p* < 0.05 and ^∗∗^*p* < 0.01, respectively. Arrows indicate the time of *L. braziliensi*s (a) and *P. yoelii* (b) infection.

Infection of BALB/c mice with 10^4^ stationary phase promastigotes of *L. amazonensis* induced chronic lesions that did not heal over time in La and La+Py groups (**Figure 4A**). During murine Malaria (from day 0 to day 25) lesions in La+Py co-infected group were significantly smaller than in La single infected group. Although lesions of La group presented greater sizes than La+Py until 28 days of infection, after that time and until the end of the experiment both groups present similar lesion sizes. Ulcerated nodules were detected first in La group (21 days post-infection) while in La+Py group visual detection occurred 1 week later (28 days post-infection) (**Figure 4B**). However, after 63 days of infection all animals from both groups presented ulcerated nodules. Limiting dilution analysis performed at the site of infection and in draining lymph nodes showed higher parasite loads in La+Py group than La single-infected group (**Figures 4C,D**).

### Co-Infection Modulate Serum Cytokine Levels Induced by *P. yoelii* 17XNL

**FIGURE 5:**
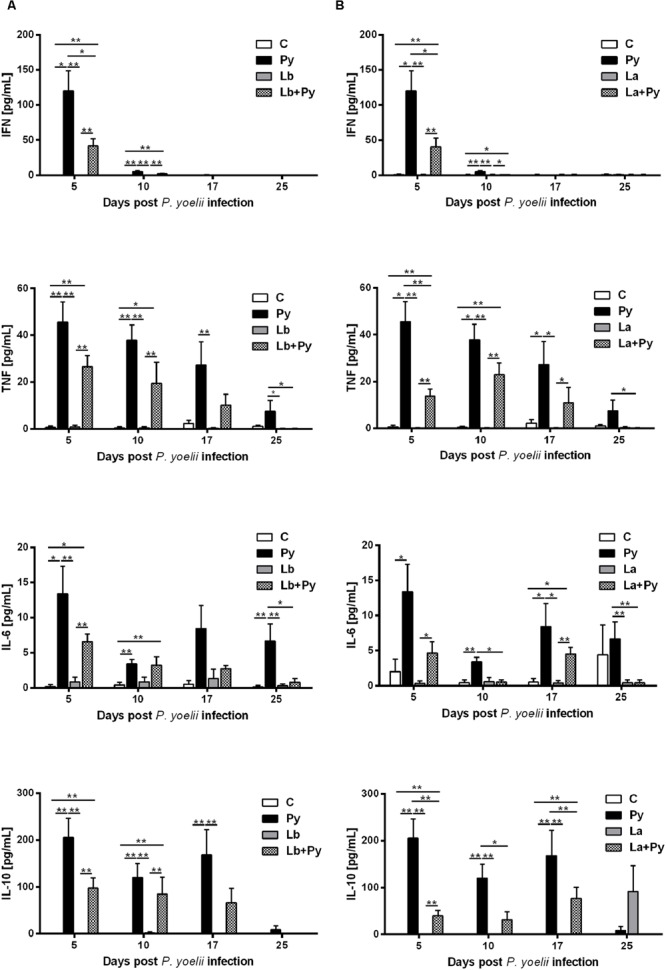
**Kinetics of cytokine levels in the serum of mice infected with *P. yoelii* (Py), *L. braziliensis* (Lb), *L. amazonensis* (La) or co-infection with *Leishmania* sp. and *P. yoelii.* Serum levels of IFN, TNF, IL-6, and IL-10 were determined by Cytokine Bead Array.** Cytokine levels obtained in the serum of non-infected controls (open bars) were compared to those obtained in Py single-infected mice (black bars) and in animals co-infected (checkered bars) or single infected (gray bars) with *L. braziliensis* **(A)** or *L. amazonensis* promastigotes **(B)**. Results are expressed by the mean ± SEM of 4–6 mice per group. The Mann–Whitney test was used to compare serum cytokine levels between groups. ^∗^*p* < 0.05 and ^∗∗^*p* < 0.01, respectively.

Cytokine levels were measured in serum samples obtained from 4 to 6 animals of each experimental group. As expected, increased levels of the proinflammatory cytokine IFN-γ, were observed in all groups infected with *P. yoelii* 17XNL (Py, Lb+Py, and La+Py) in the first week of infection (day 5). The levels of TNF, IL-6 and IL-10 also increased in the serum of the same three groups during blood stage Malaria. Cytokine levels in co-infected groups (Lb+Py and La+Py) were clearly lower than those observed in Malaria single infected group (Py) suggesting a modulatory effect of co-infection (**Figure 5**). We were not able to detect any of the analyzed cytokines in serum samples from control and Lb or La single infected animals, except for IL-10 in La infected group, only at day 25th post infection. Although we could observe a tendency for an increased production of TNF, IL-6 and IL-10 in the first 10 days of Malaria infection for Lb+Py co-infected group when compared to La+Py co-infected animals, statistic difference was observed only for IL-6 at day 10 post Py infection (*P* = 0.045). We were not able to detect the cytokines IL-2, IL-4, and IL-17 in serum samples from all experimental groups, at any time of the study.

### Recovery of Thymic Atrophy Occurs Earlier in Co-Infected Animals

**FIGURE 6:**
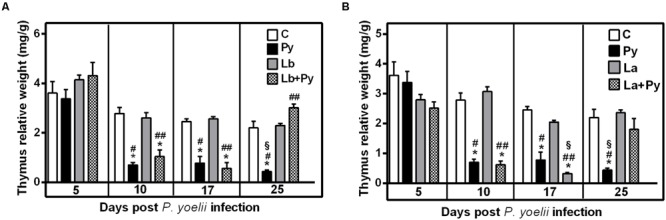
**Alterations in the thymus relative weight after infection with *P. yoelii* (Py), *L. braziliensis* (Lb), *L. amazonensis* (La), or co-infection with *Leishmania* sp. and *P. yoelii.*** Thymus relative weight in uninfected controls (open bars) were compared to those obtained in Py single-infected mice (black bars) and in animals co-infected (checkered bars) or single infected (gray bars) with *L. braziliensis* **(A)** or *L. amazonensis* promastigotes **(B)**. Results are expressed by the mean ± SEM of 4–6 mice per group. The Mann–Whitney test was used to compare thymus relative weight between groups. (^∗^) statistically different from control, (#) statistically different from *Leishmania* single infection and (§) statistically different from *Leishmania/P. yoelii* co-infected group.

In the course of Malaria infection, we observed a significant reduction of the thymus relative weight in comparison to the uninfected control group. Thymic atrophy was first observed 10 days post *P. yoelii* infection (dpi) and persisted until day 25th of infection in Py single-infected group. In co-infected groups (Lb+Py and La+Py) thymus relative weight came back to normal values at 25 day post-infection and no significant difference could be observed in comparison to the control group. During the entire experiment, *Leishmania* single-infected groups showed no differences in thymus relative weight when compared do the uninfected control group (**Figure 6**).

**FIGURE 7:**
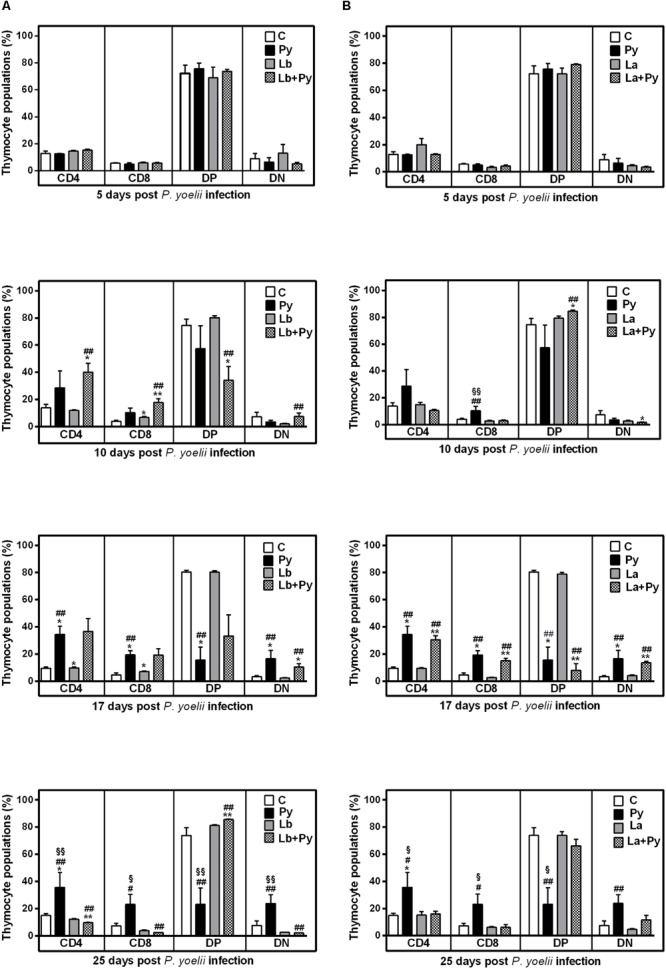
**Alterations in thymic T-cell subsets after infection with *P. yoelii* (Py), *L. braziliensis* (Lb), *L. amazonensis* (La), or co-infection with *Leishmania* sp. and *P. yoelii.* The phenotype of T subsets in the thymus were determined by flow cytometry at days 5, 10, 17, and 25 post *P. yoelii* infection.** The percentages of CD4^+^, CD8^+^, CD4^-^CD8^-^ double negatives (DN), and CD4^+^CD8^+^ double positive cells (DP) obtained in uninfected controls (open bars) were compared to those obtained in Py single-infected mice (black bars) and in animals co-infected (checkered bars) or single infected (gray bars) with *L. braziliensis.* **(A)** or *L. amazonensis* promastigotes **(B)**. Results are expressed by the mean ± SEM of 4–6 mice per group. The Mann–Whitney test was used to compare groups. (^∗^) statistically different from control, (#) statistically different from *Leishmania* sp. and (§) statistically different from *Leishmania* sp./*P. yoelii* co-infected group.

Flow cytometry analysis showed that the thymus of Py and co-infected groups underwent a decrease in CD4^+^CD8^+^ double-positive (DP) T cell subset and an increase in CD4^+^ single-positive (SP), CD8^+^SP and CD4^-^CD8^-^ double-negative (DN) T cell populations (**Figure 7**). A significant increase in CD4^+^SP thymocytes occurred at 17 dpi and persisted up to 25 dpi in Py single-infected animals. In Lb+Py group, CD4^+^SP thymocytes increased 10 days post infection, and returned to control values by day 25th post infection. In La+Py this transitory increase was observed latter at 17 dpi. The dynamics of CD8^+^SP thymocytes in single infected and co-infected groups exhibited the same profile of CD4^+^SP. When we evaluate this results together, it is possible to notice that in Lb+Py co-infection, the increase in CD4^+^SP and CD8^+^SP occurred sooner than in La+Py group (10 and 17 dpi, respectively). Double-negative thymocytes increased in Py single-infection at 17 and 25 dpi. The same increase occurred in co-infected groups only at day 17 post infection. In parallel, CD4^+^CD8^+^ DP cells significantly decreased in the thymus of Py single infected animals at 17 dpi and remained lower at 25 dpi. This decrease was observed earlier is Lb+Py group (10 dpi) but, at day 25th post infection, DP cells increased in this group and were higher than in control and single infected groups. Interestingly, in La+Py co-infected group, the increase in DP thymocytes occurred earlier (10 dpi) and was followed by a rapid decrease at day 17th post infection, coming back to control values 25 dpi. Overall, thymus recovery seems to occur faster in Malaria/*Leishmania* co-infection than in Py single infection.

### *Leishmania* Co-Infection Delay the Enhancement of Double Negative Cells Induced by *P. yoelii* 17XNL Infection in the Spleen

**FIGURE 8:**
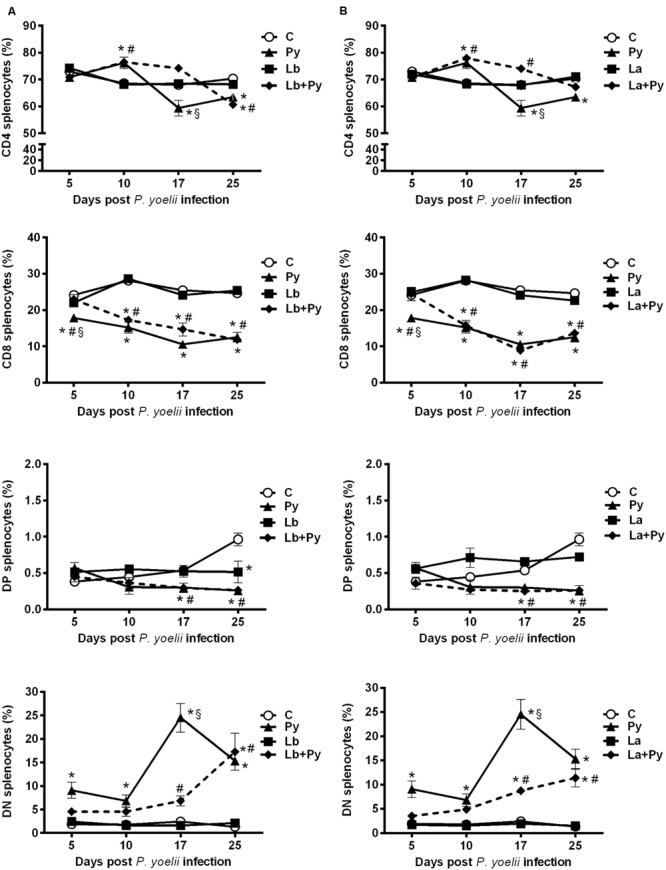
**Alterations in spleen T-cell subsets after infection with *P. yoelii* (Py), *L. braziliensis* (Lb), *L. amazonensis* (La), or co-infection with *Leishmania* sp. and *P. yoelii.* The phenotype of T subsets in the spleen were determined by flow cytometry at days 5, 10, 17, and 25 post *P. yoelii* infection.** The percentages of CD4^+^, CD8^+^, CD4^-^CD8^-^ double negatives (DN), and CD4^+^CD8^+^ double positive cells (DP) obtained in uninfected controls (open circles) were compared to those obtained in Py single-infected mice (black triangles) and in animals co-infected (black diamonds with dashed line) or single infected (black squares) with *L. braziliensis.* **(A)** or *L. amazonensis* promastigotes **(B)**. Results are expressed by the mean ± SEM of 4–6 mice per group. The Mann–Whitney test was used to compare groups. (^∗^) statistically different from control, (#) statistically different from *Leishmania* sp. and (§) statistically different from *Leishmania* sp./*P. yoelii* co-infected group.

We also evaluated the T cell phenotypes present in the spleens, mesenteric, subcutaneous and submandibular nodes (the last ones as the draining nodes from ears lesions) in single infected and co-infected animals. No alterations were observed in the T cell phenotypes present in subcutaneous and submandibular nodes among the studied groups, while in mesenteric nodes and spleens we were able to observe similar patterns of alterations that were more evident in the spleens (**Figure 8**). After a transitory increase at 10 dpi in Py single infected and in co-infected groups, CD4^+^ T cells dropped in Py single infected group at 17 dpi and remained bellow controls at 25 dpi. In Lb+Py co-infected group a significant decrease was observed only at 25 dpi. Py single infected animals also presented a significant drop in the percentages of CD8^+^T cells. In co-infected groups, the percentages of CD8^+^T cells were bellow controls at 10, 17, and 25 dpi. Low percentages of DP splenocytes were observed in Py single infected and co-infected groups, which were different form controls only when animals started to recover from malarial infection (17 and 25 dpi). On the other hand, *P. yoelii* infection increased the percentage of DN splenocytes during the entire experiment, and *Leishmania* co-infection was able to delay this enhancement. The percentages of CD4, CD8, DP, and DN splenocytes in *Leishmania* single infected groups (both Lb and La) remained similar to uninfected controls at all time points.

## Discussion

Malaria and CL are neglected diseases spread over many tropical and subtropical regions of the World showing extensive overlapping geographic distribution. In addition, the increase in travel, migration and war refugees from endemic regions are factors that also contribute to the risk of simultaneous infections by *Plasmodium* spp. and *Leishmania* spp. parasites. Some cases of human Malaria and VL co-infection were described in the past and recently in different parts of the world (Yoeli, 1948; Ab Rahman and Abdullah, 2011; van den Bogaart et al., 2012, 2014; Bin Mohanna, 2015), demonstrating that co-infection may occur more frequently than we would expect. An increasing amount of evidence shows that co-infections may affect the natural outcome and progression of diseases due to the modulation of immune response (La Flamme et al., 2002; Kolbaum et al., 2012; Qi et al., 2013; van den Bogaart et al., 2014). In the present study, we established a co-infection model of American CL and a non-lethal murine Malaria and demonstrated that co-infection is able to affect the pathogenesis and outcome of both diseases.

Pilot studies were carried out in our Laboratory in which mice were co-infected at different times and orders. It was observed that the outcome of malaria or *Leishmania* infections were the same in any time and order of infections tested. For operational reasons we selected day 3 for Py infection. In addition, our results regarding the outcome of both diseases are similar to those published by Coleman et al. (1988a,b) in *P. yoelii* and *L. amazonensis* coinfection experiments.

Rodent Malaria parasites provide great models to investigate pathogenesis and immune mechanisms during infection (Li et al., 2001; Zuzarte-Luis et al., 2014). The non-lethal strain 17XNL of *P. yoelii* generally causes a transient disease with moderate parasitaemia, weight loss, splenomegaly, hypothermia, and anemia (Coleman et al., 1988b; Kobayashi et al., 1996; Niikura et al., 2008; Kolbaum et al., 2012; Karadjian et al., 2014). In our study, BALB/c mice infected with blood-stage *P. yoelii* 17XNL develop a self-limiting infection with mild parasitaemia that resolve in 4 weeks. Prior infection with *L. braziliensis* reduced parasitaemia, while animals previously infected with *L. amazonensis* showed a tendency to have higher parasitaemia than Py single infected group. Mortality during acute Malaria increased with *L. amazonensis* co-infection, demonstrating that the existence of intrinsic differences between this two *Leishmania* species during co-infection, may affect animal survival.

The course of experimental CL is dependent on a combination of factors: *Leishmania* and host species, parasite strain, site, and size of the inoculum among other aspects (Pereira and Alves, 2008; Pereira et al., 2009; Ribeiro-Gomes et al., 2014). The murine model of subcutaneous infection with *L. major* generated a great amount of data concerning the immune response in CL. It was in this model that could be demonstrated the importance of a type 1 (proinflammatory) specific immune response for healing the infection in genetically resistant strains of mice (such as C57BL/6), as well as the dominance of a type 2 immune response (with a strong production of IL-4 and IL-10) in susceptible BALC/c mice. On the other hand, all strains of mice that have been tested so far using the subcutaneous route of infection are susceptible to *L. amazonensis*, and non-permissive hosts for *L. braziliensis*.

The intradermal rout of infection with low doses of parasites in the ears, first developed for *L. major* infection (Belkaid et al., 2000), proven also to be an important experimental model for *L. braziliensis* studies. After intradermal infection in the ears with *L. braziliensis* promastigotes BALB/c are able to develop ulcerated lesions that heal spontaneously, similar to the most common clinical manifestation of American Tegumentary Leishmaniasis caused by *L. braziliensis*: the localized cutaneous form (de Moura et al., 2005; Falcão et al., 2012; Carregaro et al., 2013). In the present work, mice infected intradermally with *L. braziliensis* developed a localized lesion that increased in size, became ulcerated and self-resolved by week 16 post infection, as expected (data not shown). Co-infected mice exhibited similar pattern in lesions development, but those were smaller and presented less ulcerations. *L. amazonensis* infection, on the other hand induced progressive non-healing lesions, and at the end of the study (11 weeks after *L. amazonensis* infection) 100% of the animals presented ulcerated lesions. Interestingly, during acute Py infection (in the 1st 4 weeks post *L. amazonensis* infection), co-infected group presented a transitory delay on the development of lesions as well as smaller lesions when compared to La single-infected mice. As mentioned before, the differences in susceptibility to *Leishmania* species in murine models, as well as in humans, have multifactorial causes, one of them being the type of immune response generated after infection. For this reason, the differences observed in the disease outcome of *L. braziliensis* and *L. amazonensis* infection during Malaria co-infection must be evaluated in the context of the intense systemic immune response elicited by *P. yoelii* 17XNL in the 1st weeks of *Leishmania* infection.

It is well described that Malaria erythrocytic stage triggers a strong IFN-γ response in both rodent and human Malaria (Shear et al., 1989; Chen et al., 2010; Perez-Mazliah and Langhorne, 2014). The first wave of proinflammatory cytokines is sustained by the innate immune system, when parasitaemia is still low, and is characterized by the release of IFN-γ, TNF-α, IL-2, and IL-12. The inflammatory environment sustains CD4^+^ T cell polarization to a Th1 phenotype that provides more IFN-γ for macrophages activation and control of parasitaemia (Artavanis-Tsakonas and Riley, 2002; Artavanis-Tsakonas et al., 2003). In our experiments, we were able to detect an early and pronounced increase of IFN-γ levels in the serum of *P. yoelii* 17XNL single infected and co-infected groups, mainly in the beginning of the infection, as extensively described in literature (Shear et al., 1989; Kobayashi et al., 1996; Shan et al., 2012; Karadjian et al., 2014). While the enhanced levels of IFN-γ in the serum was only transitory (5 and 10 days after *P. yoelii* 17XNL) the levels of TNF and IL-6 also increased in animals infected with *P. yoelii* 17XNL, but remained elevated for a longer period of time (during patent parasitaemia) in the serum. Co-infected groups presented smaller lesions than *Leishmania* single infected groups and this effect occurred simultaneously with the increased cytokines levels in the serum, during Malaria erythrocytic phase. The strong proinflammatory environment caused by *P. yoelii* 17XNL infection could explain the delay on lesions development in co-infected groups, given the fact that IFN-γ and TNF play an essential role in controlling *Leishmania* replication inside macrophages (Ashok and Acha-Orbea, 2014). Obviously a clearly scenario can be achieve if we evaluate the *Leishmania*-specific immune response developed in *Leishmania* single infected and *Leishmania* /*P. yoelii* 17XNL co-infected groups. This matter is currently under investigation.

In CL as well as in Malaria, excessive inflammatory response can lead to pathology or even death. Therefore, the balance between pro and anti-inflammatory cytokines is important to control pathology and parasitaemia (Artavanis-Tsakonas et al., 2003; Antonelli et al., 2005; Oliveira et al., 2014). Overall we could observe a tendency for reduced levels of IFN-γ, TNF, IL-6, and IL-10 in the serum of co-infected groups, when compared to *P. yoelii* 17XNL single infected group, suggesting a modulation of *P. yoelii* 17XNL induced immune response by *Leishmania* co-infection. On the other hand, *L. braziliensis* and *L. amazonensis* co-infection exerts opposite effects on *P. yoelii* 17XNL infection (**Figures 2** and **3**). IL-10 is a key regulatory cytokine to protect mice against pathology during acute Malaria (Kobayashi et al., 1996; Freitas do Rosario and Langhorne, 2012). Interestingly the reduction in the serum levels of IL-10 reached statistically significance only *in L. amazonensis* co-infected group at days 5 and 10 post *P. yoelii* 17XNL infection. This significant reduction in serum IL-10 levels could be linked to the persistent parasitaemia and deaths observed in *L. amazonensis* co-infected group (**Figure 2**).

Similar to several other parasitic diseases, experimental Malaria infections can cause severe thymic alterations (Savino, 2006; De Meis et al., 2012). During infection the organ undergoes a strong atrophy with disruption of its architecture that may influence T cell maturation. It has been already demonstrated that mice infected with *P. chabaudi* or *P. berghei*, two other rodent Malaria species, exhibit an important thymic atrophy with cellular depletion and histological disorganization (Seixas and Ostler, 2005; Andrade et al., 2008; Francelin et al., 2011; Lima et al., 2012). Thymic atrophy caused by infectious diseases may occur in combination with one or more of the following events: impairment of thymocyte proliferation, increase in thymocyte death and increase in thymocyte migration to peripheral lymphoid tissue (De Meis et al., 2012). Increase in apoptosis and thymocyte migration has been described in a variety of diseases (Savino, 2006), including Malaria (Francelin et al., 2011).

Accordingly, in *P. yoelii* 17XNL infection we observed a profound thymic atrophy with a reduction in the percentage of double positive cells (CD4^+^CD8^+^) and an increase in the percentage of single positive (CD4^+^ and CD8^+^) and double negative cells (CD4^-^CD8^-^). We also observed a high degree of apoptosis and necrosis in the thymocytes of *P. yoelii* single infected and co-infected groups, with no difference between groups (data not shown). Besides the intense atrophy during acute Malaria, thymus was capable to recover its normal size and cellularity after *P. yoelii* elimination, in accordance with observations with *P. chaubaudi* non-lethal infection (Seixas and Ostler, 2005). Infections with both *Leishmania* species were not able to cause thymus involution in mice, probably due to the localized nature of the cutaneous lesions they cause, with no or little impact in the systemic immune environment. Remarkably, thymus recovery occurred faster in co-infected animals, what we believe is a reflection of the minor inflammatory environment observed in co-infected animals.

The reduction in double positive thymocytes has also been described as common feature in infectious diseases, and can be explained by a premature escape of immature cells to blood and peripheral lymphoid organs (De Meis et al., 2012). *P. yoelii* 17XNL single infected and co-infected groups showed decreased percentages of double positive CD4^+^CD8^+^ splenocytes when compared to *Leishmania* single infected animals and uninfected controls, suggesting that the reduced percentages of double positive cells in the thymus of those groups were not due to increased migration to the peripheral lymphoid organs.

Among the mechanisms described to clear blood-stage Malaria parasites are mature isotypes antibodies and antibody-independent T cell mechanisms (Marsh and Kinyanjui, 2006; Beeson et al., 2008), but all of them require the activation of CD4^+^ T cells. In our model, CD4^+^ T cells presented a transitory increase at 10dpi but presented a significant drop on their percentages after 17 dpi when compared to controls. In *L. amazonensis* co-infected group they equalized controls only at 25 dpi, while in *L. braziliensis* co-infection they were lower them controls and *Leishmania* single infected group. The nature of this cells (e.g., if they are regulatory the cells or effector T cells), as well as the cytokines they produce after stimulation with *Leishmania* antigens or *P. yoelii* infected erythrocytes are under investigation.

Interestingly the percentages of double negatives CD4 and CD8 splenocytes increased in *P. yoelii* 17XNL infected groups, and *Leishmania* co-infection were able to delay this enhancement. We are also performing multiparametric flow cytometry experiments to better understand the phenotype of this CD3^+^CD4^-^CD8^-^cells, since they can be NKT cells or γδT cells. Both cell types are components of the innate immune system and have been proposed to play significant roles in the clearance of blood-stage malarial parasites (Stevenson and Riley, 2004; Urban et al., 2005).

NKT cells are innate-like lymphocytes that account for approximately 5% of T lymphocytes in the spleen. They posses both T cell and NK cells surface markers since they can express CD4 or CD8 co receptors on their surface, or neither one of them (double-negative phenotype) as well as the NK1.1 surface molecule (Balato et al., 2009). Primary *P. yoelii* infection with non-lethal strain 265BY is able to induce an organ-specific and heterogeneous NKT cells response (Soulard et al., 2007). Hepatic NKT cells consisted mainly of CD1d-dependent CD4^+^ and double negative NKT cells, whereas splenic NKT cells presented a CD1d-independent TCRhigh CD4high phenotype during infection (Soulard et al., 2007). It was also demonstrated that CD49b^+^ CD3^+^ natural killer T (NKT) cells increased in the liver after a primary infection with *P. yoelii* non-lethal strain (17XNL), and that CD1d-restricted NKT cells, which secrete IFNγ, are critical to reduce liver-stage burden in a secondary infection (Miller et al., 2014). Lack of type 1 IFN-alpha receptor signaling compromise the enhancement of NKT cells in the liver, showing a link between type I IFN signaling, cell recruitment, and subsequent parasite elimination.

Gamma delta T cells are the first to be generated in the ontogeny. They are able to respond quickly through the production of cytokines and can be divided into two types: interferon-producing cells (CD27hi) and IL-17-producing cells (CD27lo) (Zarin et al., 2015). In Malaria they are activated during pre-erythrocytic (liver) and erythrocyte stages and can produce INF-γ *in vitro* after stimulation with *P. falciparum* infected erythrocytes (Elloso et al., 1994; Farouk et al., 2004; Scholzen and Sauerwein, 2016). In BALB/c mice, the number of γδT cells increases considerably in the spleen during infection with *P. yoelii* non-lethal strain (17XNL) (Kopacz and Kumar, 1999), while this increase did not occur to the same extent in mice infected with the lethal strain of *P. yoelii* (17XL) suggesting that γδT cells play a protective role in Malaria (Kopacz and Kumar, 1999). In another work with *P. yoelii* non-lethal strain was observed an increase in the percentage of γδT cells in the spleen, liver and peripheral blood, peaking at day 21 of infection (Li et al., 2012). When the mice were depleted from γδT cells with monoclonal antibodies parasite elimination was delayed (Li et al., 2012).

Our results demonstrating an increase in CD3^+^CD4^-^CD8^-^ cells during erythrocyte stage in *P. yoelii* 17XNL non-lethal strain infected animals both in the thymus (**Figure 7**) and in the spleen (**Figure 8**) does not discard the possibility that those can be either NK T or γδT cells, and the delay of this enhancement to occur in *Leishmania* co-infected groups when compared to *P. yoelii* single infected animals could have a correlation with the differences in cytokines production observed between *P. yoelii* 17XNL single infected and co-infected groups.

Taken together, our results suggest that coexisting infection with *P. yoelii* 17XNL and *L. braziliensis* or *L. amazonensis* may change diseases outcomes, and that Malaria outcome can be altered according to the *Leishmania* co-infection specie evaluated. On the other hand Malaria infection produced a transient delay on leishmanial lesions development. These alterations on Malaria and CL progress seem to be closely related to changes in the immune response as verified by alteration in serum cytokine levels and thymus dynamics during infection.

In areas where Malaria and CL are co-endemic, human populations are continuously exposed to Malaria and Leishmaniasis vectors bites and consequently are at risk of suffering repeated infections. These recurrent infections in endemic areas might cause permanent immunomodulatory effects, which can probably interfere in the outcome of both diseases. Moreover, the increase in Malaria parasitaemia, patency and mortality, observed in *L. amazonensis/P. yoelii* coinfection, might contribute to the severity of disease as well as to the maintenance of the malaria transmission in co-endemic areas. Considering the importance and the geographical distribution of Malaria and CL in tropical regions of the world, further investigation on immunomodulatory mechanisms during co-infection are necessary to understand how co-infection can affect not only the natural history and progression of diseases but also the treatment and prevention of both.

## Author Contributions

Conceived and designed the experiments: DB, PL, JF, and RP. Performed the experiments: RP, PL, DS, DV, DP, and DB. Analyzed the data: RP, PL, DV, DS, and DB. Contributed reagents/materials/analysis tools: DB, PL, and DV. Wrote the paper: RP, PL, and DB.

## Conflict of Interest Statement

The authors declare that the research was conducted in the absence of any commercial or financial relationships that could be construed as a potential conflict of interest.
